# Allogeneic Platelet Releasate Preparations Derived via a Novel Rapid Thrombin Activation Process Promote Rapid Growth and Increased BMP-2 and BMP-4 Expression in Human Adipose-Derived Stem Cells

**DOI:** 10.1155/2016/7183734

**Published:** 2015-12-28

**Authors:** Michael McLaughlin, Paul Gagnet, Elizabeth Cunningham, Randi Yeager, Michael D'Amico, Katie Guski, Michael Scarpone, Daniel Kuebler

**Affiliations:** ^1^Department of Biology, Franciscan University of Steubenville, Steubenville, OH 43952, USA; ^2^Trinity Sports Medicine and Performance Center, Trinity Hospital, Steubenville, OH 43952, USA

## Abstract

The administration of human adipose-derived stem cells (ASCs) represents a promising regenerative therapy for the treatment of orthopedic injuries. While ASCs can be easily isolated from liposuction-derived adipose tissue, most clinical applications will likely require *in vitro* culture expansion of these cells using nonxenogeneic components. In this study, platelet releasate was generated using a novel rapid thrombin activation method (tPR). ASCs grown in media supplemented with tPR proliferated much faster than ASCs grown in media supplemented with 10% fetal bovine serum. The cells also retained the ability to differentiate along chondrogenic, adipogenic, and osteogenic lineages. The tPR cultured ASCs displayed elevated expression of BMP-4 (5.7 ± 0.97-fold increase) and BMP-2 (4.7 ± 1.3-fold increase) and decreased expression of PDGF-B (4.0 ± 1.4-fold decrease) and FGF-2 (33 ± 9.0-fold decrease). No significant changes in expression were seen with TGF-*β* and VEGF. This pattern of gene expression was consistent across different allogeneic tPR samples and different ASC lines. The use of allogeneic rapidly activated tPR to culture ASCs is associated with both an increased cell yield and a defined gene expression profile making it an attractive option for cell expansion prior to cell-based therapy for orthopedic applications.

## 1. **Introduction**

Given their therapeutic potential and ease of isolation, there is widespread interest in using human adipose-derived stem cells (ASCs) to treat a variety of musculoskeletal and joint disorders [1–3]. These cells can be easily isolated from subcutaneous adipose tissue, they can differentiate into adipocytes, osteocytes, chondrocytes, and tenocytes, and they secrete a variety of growth factors that can aid in tissue repair [4–6].

Standard isolation protocols involve enzymatic digestion of lipoaspirates to isolate the stromal vascular fraction (SVF) [4]. The SVF is a heterogenous collection of cells, of which only a small fraction (roughly 1–5%) are ASCs [7, 8]. While limited amounts of ASCs can be isolated directly from the lipoaspirate, subsequent culturing of the plastic adherent cells in the SVF can yield large numbers of ASCs.

Preliminary studies in humans have demonstrated that SVF injections have the ability to treat cartilage and bone defects while maintaining an excellent safety profile [9–12]. However, given the limited number of ASCs in the SVF, the use of culture expanded cells will likely be necessary to achieve optimal therapeutic results. In fact, pilot studies with cultured ASCs and bone marrow derived stromal stem cells have shown promising results in treating bone and joint disorders [13–17].

As a result, the identification of safe, reliable, and robust cell culturing protocols that do not hinder the therapeutic potential of these cells remains an important factor to optimize before ASC treatments for orthopedic disorders become widespread. In basic research, fetal bovine serum (FBS) is commonly used as a media supplement for culturing ASCs, but, given the variability in FBS preparations and the potential for xenogeneic contamination, FBS substitutes are essential for culturing cells for specific clinical applications [18, 19].

Previous research has demonstrated that thrombin-activated platelet releasate (tPR) and platelet lysate (PL) are effective FBS substitutes for culturing mesenchymal stem cells (MSCs) as both have been shown to increase the growth rate of MSCs in culture [18, 19]. While both augment growth, the two preparations contain different subsets of growth factors and small molecules due to the different manners in which they are prepared [20]. While PL preparations liberate the entire contents of the platelets and contain aggregates of platelet membranes, thrombin activation triggers a burst of growth factor release that mimics what occurs* in vivo* during wound healing and tissue repair [18]. As a result, tPR preparations may have therapeutic advantages over PL preparations.

Following activation, platelets release a variety of growth factors that can affect ASC growth and differentiation including platelet derived growth factor B (PDGF-B), insulin-like growth factor-1 (IGF-1), transforming growth factor beta (TGF-beta), vascular endothelial growth factor (VEGF), and basic fibroblast growth factor (FGF-2) [21]. However, as platelet counts and the level of platelet secreted growth factors vary from person to person [22, 23], it is not clear how generally applicable ASC culture using tPR will be in a clinical setting. To address this, it will be necessary to examine the effects a variety of tPR preps have on the growth and gene expression of different ASC lines.

Given the benefits of using early passage cells in autologous treatments in the clinic—the reduced time between harvesting adipose tissue and patient treatment and the reduced possibility of contamination and spontaneous transformation during the culturing process—studies on early passage cells would be most relevant for clinical usage.

In the present study, we generated a variety of allogeneic tPR preparations via a novel rapid thrombin activation protocol. We examined the effect these allogeneic tPR preparations had on early passage ASCs by investigating (1) the ability of tPR to rapidly produce large numbers of ASCs competent for therapeutic use and (2) the inherent variability different allogeneic tPR preps had on these early passage cells. We examined the effect tPR had on the growth rate, morphology, gene expression, and differentiation ability of ASCs to determine tPR's suitability in short-term culturing of ASCs for use in orthopedic therapies.

## 2. **Methods**

*ASC Isolation*. ASCs were isolated from liposuction aspirates harvested from subcutaneous adipose tissue sites of subjects undergoing orthopedic procedures at the Trinity Sports Medicine and Performance Center Clinic. The research protocol used was approved by the Franciscan University of Steubenville Institutional Review Board. To isolate the ASCs, lipoaspirate samples were washed repeatedly in a syringe using HBSS. After washing, adipose tissue was digested with 0.1% collagenase (type I; Worthington) in a 37°C water bath for 1 hr with gentle agitation. The digest was then centrifuged for 5 minutes at 500 g to pellet the stromal vascular fraction (SVF). The SVF was resuspended in HBSS and passed through a 40-micron filter. The SVF was repelleted by centrifuging for 5 minutes at 500 g. The cells were resuspended in appropriate growth media and the live nucleated cells were counted on a Nexcelom Cellometer using an AO/PI dye.

*Platelet Isolation and Rapid Thrombin Activation*. Platelets were isolated from peripheral blood from healthy adult donors using the Harvest SmartPrep System. The APC-60 version was used to prepare 5–10 mLs of platelet rich plasma (PRP) per donor. Platelet counts for the four samples as determined by a Coulter Counter (Beckman Coulter) were as follows: sample 1 = 6.2 × 10^5^/*μ*L, sample 2 = 7.3 × 10^5^/*μ*L, sample 3 = 5.6 × 10^5^/*μ*L, and sample 4 = 10.0 × 10^5^/*μ*L. To rapidly generate thrombin-activated platelet releasate (tPR), 5 mLs of concentrated platelets was activated by 0.5 mLs of thrombin (Recothrom, 1000 IU/mL) for 2-3 minutes at room temperature with gentle agitation. The activated platelets were immediately centrifuged at 10,000 g and the supernatant was isolated and stored at −80°C until use. Prior to use, the tPR was warmed to room temperature, centrifuged at 10,000 g to remove any residual clotting debris, and added directly to the culture media as described below. Heparin was not added to the growth media as in previous studies [18] as (1) heparin is known to reduce cell growth in ASCs [18] and (2) we did not observe gel or clot formation during cell culture with our rapidly activated tPR.

*Cell Culture*. Freshly isolated SVF cells were plated at a density of 4000 live nucleated cells/cm^2^ in 25 cm^2^ flasks. These passage 0 (P0) cells were grown in DMEM/F12 media (GIBCO) supplemented with either 10% FBS or 10% tPR. Cells were cultured for 10 days with the media being changed twice. On day 10, the cells were trypsinized, counted using a Nexcelom Cellometer Vision CBA Cytometry System, and seeded at a density of 4000 cells/cm^2^ in DMEM/F12 media supplemented with either 10% FBS or 10% platelet releasate. These P1 cells were cultured for 4 days with the media being changed on day 2.

*Immunophenotyping*. Passage 2 tPR cultured ASCs were characterized for the expression of the following markers: CD34 (single-pass adhesion molecule), CD73 (ecto-5′ nuclease), and CD90 (THY-1). Cells were detached using trypsin, washed in HBBS, and stained using specific FITC-conjugated monoclonal antibodies or isotype-matched controls (BioLegend). Analysis was performed on a Nexcelom Cellometer Vision CBA Cytometry System. Cultured mesenchymal stem cells are positive for CD73 and CD90 and are negative for CD34.

*Real-Time PCR*. Passage 1 cells were trypsinized following exposure to the indicated culturing conditions and total RNA was extracted using the BioRad total Arum RNA prep kit and on-column DNase digestion to eliminate genomic DNA. Quantity and quality of the total RNA for each preparation were determined using a Nanoquant plate on a Tecan 2000 spectrophotometer. RNA from each sample (~0.2 mg) was reverse-transcribed using iScript RT (Bio-Rad). For quantitative real-time PCR, reactions were performed using a SYBR green PCR kit (BioRad) and a MJ Mini thermal cycler (BioRad). All reactions were carried out using validated primer pairs purchased from http://realtimeprimers.com/. All rt-PCR reactions were carried out in duplicate and control rt-PCR reactions were performed using templates that had been mock reversed-transcribed (no reverse transcriptase). The control reactions confirmed that genomic DNA had been efficiently removed. A ΔΔCq method (BioRad) was then used to calculate relative gene expression using GAPDH and RP13L as reference genes. Previous work has identified these reference genes as being stably expressed in ASCs following calcium chloride activated PRP and FBS culture [24].

*Cell Differentiation*. For adipocyte and osteocyte differentiation, passage 2 ASCs grown in tPR were harvested and seeded at a density of 5000 cells/cm^2^ in 6-well dishes. After cells reached sixty percent confluency, differentiation media were added. Adipocyte differentiation media consisted of standard media supplemented with 1 *μ*m dexamethasone, 0.5 mM isobutylmethylxanthine, 100 *μ*m indomethacin, and 20 *μ*g/mL insulin. After three weeks, the cells were stained using Oil Red O as an indicator of intracellular lipid accumulation.

For osteocyte differentiation, StemPro osteogenesis media (GIBCO) was used and changed every three days. After 21 days, the cultures were stained using Alizarin Red, which dyes calcium deposits bright orange-red.

For chondrocyte differentiation, micromass cultures were generated by centrifuging passage 2 ASCs and resuspending them in standard growth media at a concentration of 1 × 10^7^ cells/mL. 10 *μ*L droplets were seeded on wells of a 24-well plate and incubated for 12–15 hours under high humidity. After the micromass had formed and attached to the well, 0.5 mLs of warmed StemPro chondrogenesis media (GIBCO) was added. The media were changed every three days. After 21 days, the micromass pellet was rinsed once with HBSS and fixed with 4% formaldehyde solution for 30 minutes. The pellet was rinsed again and stained for 30 minutes with 1% Alcian Blue solution prepared in 0.1 N HCl. The pellet was rinsed three times with 0.1 N HCl and once with dH_2_O. Blue staining indicates synthesis of proteoglycans by chondrocytes.

## 3. Results

### 3.1. Morphology

**Figure 1:**
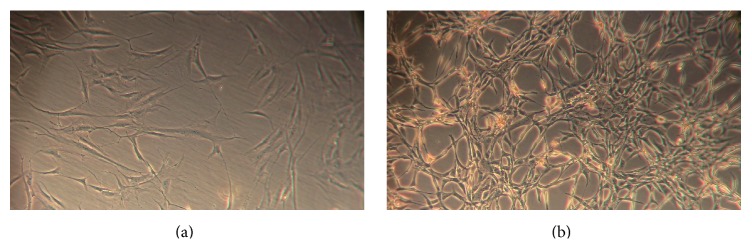
Morphological changes in passage 1 ASCs grown in tPR. 100,000 passage 1 ASCs were plated in T25 flasks and grown for 4 days in either 10% FBS or 10% tPR. (a) Morphology and density of FBS cultured cells after 4 days. (b) Morphology and density of tPR cultured cells after 4 days.

Freshly isolated P0 and P1 ASCs were cultured in DMEM media supplemented with either 10% FBS or 10% tPR. ASCs cultured under both conditions displayed the characteristic ASC fibroblast morphology, although the tPR cultured cells were smaller and more spindle-like in shape (Figure 1). This alteration in cell morphology was consistent across all four allogeneic preparations of tPR and was maintained through multiple passages.

In addition to changes in morphology, cells cultured in tPR displayed a reduced level of adhesion to the culture dish as previously reported [18]. Cells grown in tPR detached from the plastic dish within 2–4 minutes of the onset of trypsin addition, while FBS cultured cells required longer incubation times, 5–8 minutes, to completely detach.

### 3.2. Growth Rate

**Figure 2:**
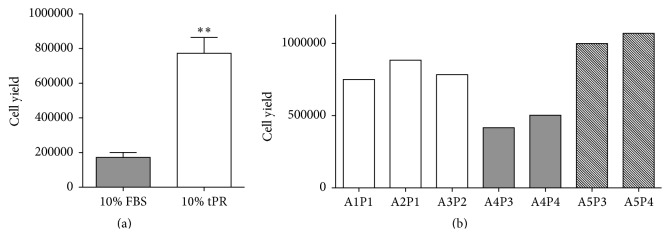
The effects of tPR on the growth of passage 0 ASCs. 100,000 freshly isolated SVF cells were plated and grown for ten days in either 10% FBS or 10% PR. (a) tPR treated cells yielded over four times as many cells after ten days in culture (*P* < 0.01). (b) Various ASC lines at P0 (A1–A5) were treated with different tPR preps (P1–P4) to determine the variability different cell lines and tPR preps would have on cell growth. The variability seen in the P0 yields appears to be attributable to differences in the adipose tissue samples.

We examined the growth rate of early passage ASCs when cultured with 10% tPR. After plating 100,000 nucleated cells from the SVF in T25 flasks, tPR treated cells proliferated at a faster rate compared to FBS supplemented cells. After ten days of growth, tPR supplemented cells yielded 4.5 times as many cells (Figure 2(a)). While there was some variability in the magnitude of the effect (3- to 7.5-fold increase), all four allogeneic tPR preps increased the yield of P0 ASCs.

To examine whether this variability could be attributed to different ASC cell lines or different tPR preps, we examined growth using various combinations of ASC lines (A1–A5) and PR preps (P1–P4) (Figure 2(b)).

The variability in the P0 yields appears to be attributable to differences in the adipose tissue samples. For example, adipose tissue sample A4 gave very similar cell yields when treated with two different PR preps, P3 and P4 (Figure 2(b)). A similar pattern was also seen with adipose tissue sample A5 (Figure 2(b)).

**Figure 3:**
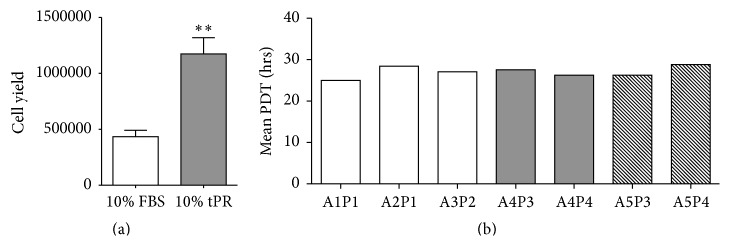
The effects of tPR on the growth of passage 1 ASCs. (a) 100,000 passage 0 ASCs were passaged and grown for 4 days in either 10% FBS or 10% tPR. Starting from an initial plating of 100,000 cells, tPR supplemented media displayed a significantly higher cell yield after four days (*P* < 0.01). (b) Little variability was seen in the population doubling times (PDT) in various ASC lines (A1–A5) exposed to different tPR preps (P1–P4). In particular, cell lines A4 and A5 were cultured in both P3 and tP4 PR preps and no significant differences in growth were seen.

P1 ASCs also displayed higher growth rates when cultured with 10% tPR. From an initial plating of 100,000 cells, four days in 10% tPR yielded roughly 2.5 times as many cells as did the 10% FBS cultures (Figure 3(a)). The cell yield was remarkably consistent across different ASC lines (A1–A5) and different allogeneic tPR preparations (P1–P4) as all seven different culture combinations yielded between 1.0 × 10^6^ and 1.5 × 10^6^ cells (Figure 3(b)).

Based on the growth rate for both P0 and P1 ASCs in tPR seen here, 1 cc of collagenase digested adipose tissue—which we find to typically yield roughly 400,000 nucleated cells—could generate over 3 × 10^7^ ASCs in just 14 days.

### 3.3. Differentiation Capacity

The differentiation potential of ASCs grown in tPR was tested to determine if these cells maintained their ability to differentiate along osteogenic, chondrogenic, and adipogenic lineages under standard* in vitro* differentiating conditions.

**Figure 4:**
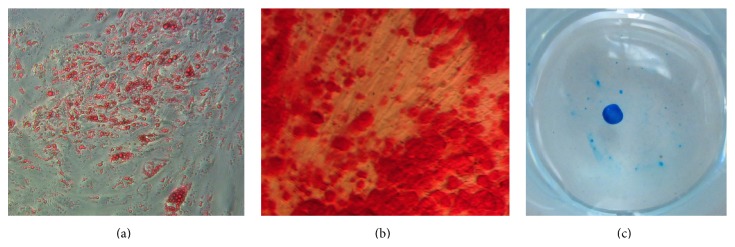
Differentiation of tPR cultured ASCs along adipogenic, osteogenic, and chondrogenic lineages. Passage 2 ASCs grown in 10% tPR still retained the ability to differentiate into adipocytes ((a) Oil Red O), osteocytes ((b) Alizarin red), and chondrocytes in a micromass culture ((c) Alcian blue).

Using passage 2 tPR cultured ASCs, we demonstrated that early passages in tPR supplemented media did not adversely affect the differentiation potential of ASCs. Osteogenic differentiation was verified by deposition of a calcium-rich matrix using Alizarin red staining. Adipogenic differentiation was verified by the formation of lipid vacuoles using Oil Red O staining (Figure 4). Finally, chondrogenic differentiation was verified in high-density micromass cultures. These micromass cultures stained positive for Alcian blue, which is specific for the highly sulfated proteoglycans of cartilage matrices (Figure 4).

**Figure 5:**
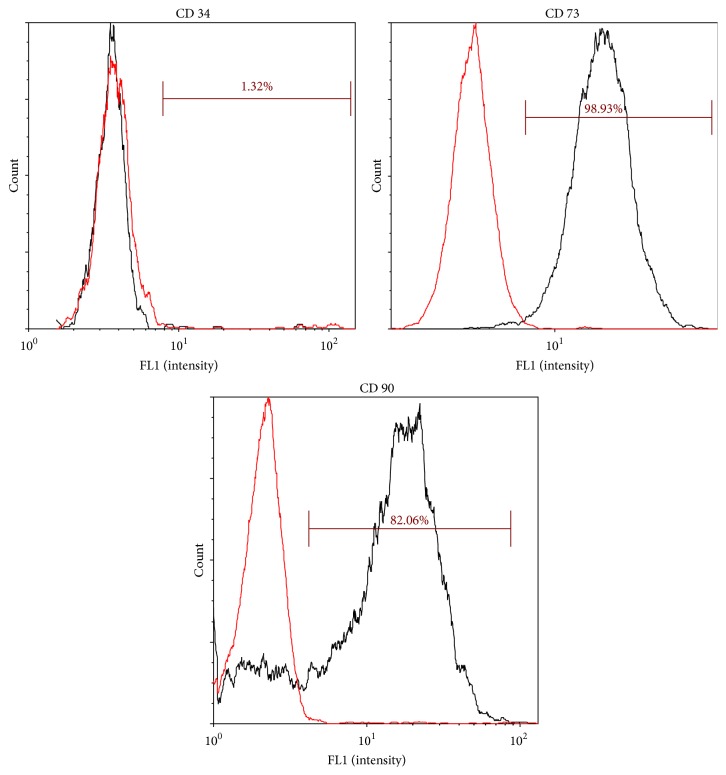
Immunophenotypical characterization of ASCs cultured in tPR. Passage 2 ASCs grown in tPR were positive for CD73 and CD90, mesenchymal stem cell markers, and were negative for CD34, a hematopoietic stem cell marker not typically found on passaged mesenchymal stem cells [25]. Black and red histograms represent specific and control antibody staining, respectively.

In addition to maintaining normal ASC differentiation potentials, passage 2 tPR cultured ASCs displayed an immunophenotype consistent with mesenchymal stem cells. These cells had intense positive expression of CD70 and CD93, both of which are MSC cell surface markers, while being negative for the hematopoietic stem cell marker CD34 (Figure 5).

### 3.4. Gene Expression

In order to examine the effect tPR culture had on the gene expression of ASCs, RT-PCR analysis was performed on a set of growth and differentiation associated genes. P1 cells that had grown to ~80% confluence were tested and four independent tPR samples were used. The six genes that were tested were bone morphogenetic protein 2 (BMP-2), bone morphogenetic protein 4 (BMP-4), transforming growth factor-beta (TGF-beta), vascular endothelial growth factor (VEGF), platelet derived growth factor B (PDGF-B), and basic fibroblast growth factor (FGF-2).

**Figure 6:**
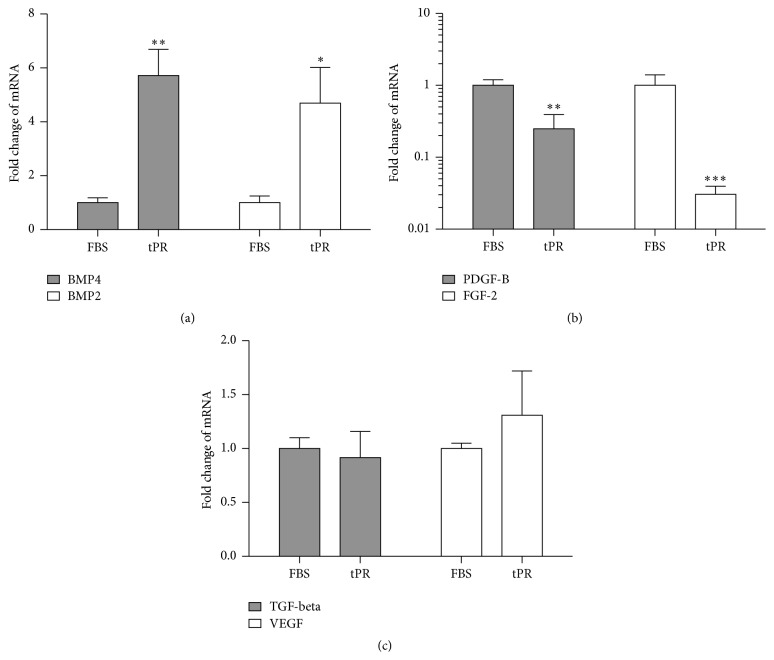
Gene expression in passage 1 ASCs grown in tPR. (a) tPR treated cells displayed an increase in BMP-2 and BMP-4 expression as compared to cells grown in FBS. (b) tPR treated cells displayed a decrease in PDGF-B and FGF-2 expression as compared to cells grown in FBS. (c) Expression levels of VEGF and TGF-beta did not change. RNA was isolated from cultured cells using the BioRad Aurum minikit and rt-PCR was performed using GAPDH and RP13A as reference genes (^*∗*^ *P* < 0.05, ^*∗∗*^ *P* < 0.01, and ^*∗∗∗*^ *P* < 0.001).

The expression of BMP-2 (4.7 ± 1.3-fold increase) and BMP-4 (5.7 ± 0.97-fold increase) was upregulated in ASC cultures grown in tPR while the expression of PDGF-B (4.0 ± 1.4-fold decrease) and FGF-2 (33 ± 9.0-fold decrease) was downregulated in tPR. While consistent changes were seen in these four factors in all four tPR preps, the expression of TGF-beta and VEGF in ASCs was not significantly altered by culture in tPR (Figure 6).

**Figure 7:**
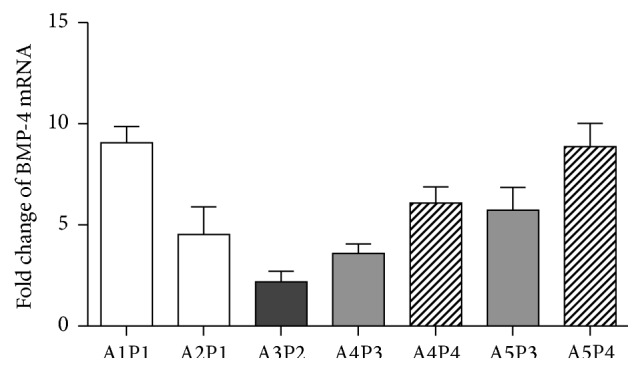
Consistent changes in BMP-4 expression in various ASCs lines in response to tPR. Changes in BMP-4 expression were relatively consistent across various ASC lines (A1–A5) exposed to different tPR preps (P1–P4). A similar pattern was seen with the other genes examined in this study. While certain PR preps, P4 in particular, tended to increase BMP-4 expression to a greater extent than others (P3), all preps increased expression.

While the magnitude of the changes in gene expression varied from sample to sample, the directionality of the changes in gene expression was consistent across all the various tPR preps and ASC lines as illustrated for BMP-4 in Figure 7.

## 4. Discussion

Culture-based expansion of ASCs will be an essential step in a variety of stem cell-based therapies, and the specific culture environment is likely to have an effect on therapeutic outcomes. Given the potential for xenogeneic contamination, a variety of human-based FBS alternatives have been reported including the use of human tPR [18–20, 26].

The increase in cell proliferation seen here using tPR is consistent with other reports that examined the response of cultured ASCs to various preparations of platelet lysate (PL) or tPR [18–20, 26]. While PL versus tPR has similar effects on ASC growth, tPR appears to be the more desirable alternative for a number of reasons. First, the thrombin activation of platelets mimics the normal physiological response of platelets during the healing process* in vivo* and can be done extremely rapidly (3–5-minute activation time) in the method described here. Secondly, it is easier to separate the platelets from platelet-factor rich supernatant than it is to separate the membrane fragments generated during platelet lysis [18].

While tPR has been shown to be effective in increasing cell proliferation, little is known about the variable effects different tPR preps have on ASCs. Previous studies have typically pooled platelets from multiple sources to produce tPR and have not looked at variability between individual preparations [18, 26, 27]. In the case of PL, one previous study examined the variable effects on growth using different PL preparations generated by a freeze/thawing protocol and found that all PL preps increased cell proliferation but to varying degrees [28].

Here we have shown that four different allogeneic tPR preps, all generated by a novel rapid thrombin activation process, exhibited consistent effects on ASCs. These four different tPR preps had platelet concentrations that ranged in 5.6–10.0 × 10^5^/*μ*L. Despite these differences in platelet concentration, all four tPR preps had very similar effects on the behavior of ASCs. These effects included an increase in cell proliferation, the maintenance of differentiation potential, the alteration of cell morphology, and consistent alterations on gene expression.

The different tPR preps had very similar effects on the growth of ASC lines. This was particularly evident during passage 1 where the cell yield ranged from 1 to 1.5 million cells following four days of culture from an initial seeding of 100,000 cells. Interestingly, there was no correlation between the growth rate and the number of platelets in the various tPR preps. However, given the variation in platelet quality between individuals [22, 23], it is not surprising that there was no direct correlation between platelet number and growth enhancement.

There was significant variation in the growth of P0s, but this variability also did not correlate with platelet levels in tPR samples. The variability seen here in P0 yields most likely stems from the inherent variability in the number of ASCs isolated from different adipose tissue samples. Support for this can be seen in ASC prep 3, which displayed very similar P0 yields when grown in two different tPR preps. A nearly identical pattern was seen with ASC prep 4.

While the proliferation promoting effects of ASCs in tPR has been well studied, less is known regarding the effect tPR has on gene expression. To our knowledge, only one previous study has examined gene expression in ASCs following tPR exposure [27]. This study used a microarray-based assay and found 102 genes with altered levels of expression. The genes they found to be altered, mostly differentiation and cell adhesion associated genes, displayed overlap with genes previous studies found to be altered in ASCs and BMSCs cultured in human serum or platelet lysate [29, 30].

We chose to analyze six genes that are associated with mesenchymal stem cell proliferation and differentiation and found that four of them had altered expression in tPR. These four genes, PDGF and FGF (downregulated) and BMP-2 and BMP-4 (upregulated), were not identified in the previous microarray-based study [27]. There are a number of factors that could have contributed to this difference. The first is the fact that the previous study examined gene expression in P2 cells while, in the present study, gene expression was examined in P1 cells. In addition, the rapid tPR prep employed here mitigated the need to add heparin, a factor that has an inhibitory effect on cell growth [18], to the culture media as in the previous study. Finally, the increased sensitivity of the RT-PCR protocol employed here may have identified differences that would be missed in a microarray-based assay.

Because the tPR from the previous study was generated from a pooled sample of eight donors [27], intersample tPR variability on gene expression could not be determined. The fact that we generated individual tPR preparations allowed us to examine the effect of intersample variability on gene expression. Similar to the changes we saw in growth rates, we found that the changes in gene expression were consistent across all four tPR preps tested.

While the effect these changes have on the therapeutic potential of these cells is untested, the consistent increase in BMP-2 and BMP-4 expression may be beneficial to the repair of both bone and cartilage tissue. BMP-2 has been shown to increase proliferation and cartilaginous ECM production in MSCs [31, 32]. Likewise, BMP-4 has been shown to (1) induce differentiation of MSCs into mature chondrocytes and (2) enhance the production of cartilaginous ECM by stimulating synthesis of collagen type II and aggrecan [31, 33]. In the case of bone repair, the delivery of BMP-2 has been shown to augment the ability of ASCs to differentiate into osteocytes and to augment bone repair [34, 35].

The reduction in PDGF-B and FGF-2 expression in tPR, while being likely not optimal for tissue repair [31], is not surprising given the high amounts of these growth factors typically found in PR preps [19]. In fact, neutralization of FGF alone or in combination with PDGF has been shown to reduce the proliferative effect of PL preparations [36]. Therapeutically, the reduction in PDGF and FGF expression could be offset through the coinjection of tPR cultured ASCs along with platelet rich plasma (PRP). Such an approach has shown promise in both human and animal studies of articular cartilage damage [9, 37, 38].

Given that PRP and subsequent tPR preps contain a wide variety of growth factors, proteins, peptides, and other biologically relevant small molecules, it will be difficult to identify all the factors that are important to the growth modifying effects of tPR seen here and elsewhere [18, 21, 26, 27]. Despite this, the speed and ease by which allogeneic tPR preps described here can be produced, coupled with their consist beneficial effects on cell growth, make tPR an ideal culture supplement for the generation of adequate numbers of ASCs for orthopedic therapies.

Based on the growth rate for ASCs in tPR seen here, 1 cc of collagenase-digested adipose tissue could yield over 3 × 10^7^ ASCs in just 14 days. This number of ASCs is at or above the therapeutic MSC cell dosages found to be efficacious for cartilage and bone repair in various pilot studies [13–17]. In addition, this yield could easily be scaled up via the harvesting of additional adipose tissue.

Finally, the results here demonstrate that the initial growth and proliferation of ASCs were more dependent on the adipose tissue sample than on the tPR prep. Likewise, the magnitude of the changes in gene expression varied depending upon the specific tissue sample. Clinically, this finding indicates that the health of the patient may be an important factor affecting the growth, proliferation, cytokine secretion, and ultimately the therapeutic potential of the autologous ASCs. In fact, factors such as age, gender, body mass index, and disease state all can affect the function and behavior of a patient's ASCs [39–41]. Addressing these issues prior to cell harvesting and treatment will be important in obtaining the best functional outcomes for patients involved in ASC-based orthopedic therapies.

## 5. Conclusions

The use of allogeneic rapidly activated tPR to culture ASCs is associated with both an increased cell yield and maintenance of differentiation potential. The increased cell grown will facilitate a reduced time between harvesting adipose tissue and patient treatment and the reduced possibility of contamination and spontaneous transformation during the culturing process. In addition, tPR cultured cells displayed a defined gene expression profile in response to various allogeneic tPR preps that included the upregulation of BMP-2 and BMP-4. The consistent effects of a variety of allogeneic tPR preps on different ASC lines indicate that tPR culturing is a generally applicable option for cell expansion prior to cell-based therapy for orthopedic applications.
